# PTSD prevalence and factors in intern nursing students after COVID-19 full liberalization in China: a cross-sectional study

**DOI:** 10.3389/fpubh.2024.1374977

**Published:** 2024-03-15

**Authors:** Yuanhao Sun, Xiangdong Li, Hairong Liu, Xiaoping Li, Lu Sun, Lin Zhang, Congzhi Wang, Jing Li, Mingming Liu, Dongmei Zhang, Yunxiao Lei, Ting Yuan

**Affiliations:** ^1^Department of Graduate School, Wannan Medical College, Wuhu, China; ^2^Department of Gerontology, Yijishan Hospital, the First Affiliated Hospital of Wannan Medical College, Wuhu, China; ^3^School of Humanities and Management Science, Wannan Medical College, Wuhu, China; ^4^Anhui Key Laboratory of Philosophy and Social Sciences for Public Health Crisis Management, Wuhu, China; ^5^Department of Emergency and Critical Care Nursing, School of Nursing, Wannan Medical College, Wuhu, China; ^6^Department of Internal Medicine Nursing, School of Nursing, Wannan Medical College, Wuhu, China; ^7^Department of Surgery Nursing, School of Nursing, Wannan Medical College, Wuhu, China; ^8^Department of Pediatric Nursing, School of Nursing, Wannan Medical College, Wuhu, China; ^9^Department of Gynecology and Obstetrics Nursing, School of Nursing, Wannan Medical College, Wuhu, China

**Keywords:** post-traumatic stress disorder, resilience, fear, intern nursing students, COVID-19, China

## Abstract

**Objective:**

This study aimed to assess the prevalence of post-traumatic stress disorder (PTSD) and its influencing factors among intern nursing students after the full liberalization of the COVID-19 prevention and control policy in China.

**Methods:**

Participants completed the online survey from January 14 to January 19, 2023. A demographic questionnaire, COVID-19 and internship-related questionnaire, the Fear of COVID-19 scale, the Primary Care PTSD Screen, and the Connor-Davidson Resilience Scale were used to conduct the online survey.

**Results:**

Of 438 participants, 88.4% tested positive for COVID-19 in the last 6 months. The prevalence of fear, resilience, and PTSD was 16.9, 15.5, and 11.2%, respectively. Direct care of COVID patients in hospital (OR = 2.084, 95%CI 1.034 ~ 4.202), the experience of occupational exposure (OR = 2.856, 95%CI 1.436 ~ 5.681), working with an experienced team (OR = 2.120, 95%CI 1.070 ~ 4.198), and fear COVID-19 (OR = 8.269, 95%CI 4.150 ~ 16.479) were significantly and positively associated with PTSD in nursing internship students.

**Conclusion:**

After COVID-19 full liberalization in China, intern nursing students still experienced pandemic-related mental distress, which can bring PTSD. Adequate support and counseling should be provided, as needed, to intern nursing students who are about to enter the workforce and have experienced severe PTSD symptoms related to COVID-19. Our findings indicated that should understand the importance of screening, formulate intervention strategies and preventive measures to address psychosocial problems, and provide coping skills training to intern nursing students.

## 1 Introduction

The Coronavirus disease 2019 (COVID-19) was labeled as a pandemic by the World Health Organization (1), in March 2020. With highly contagious, morbidity and mortality, COVID-19 has posed numerous challenges to all aspects of life and health. From the beginning of the COVID-19 pandemic through the fall of 2022, the Chinese government adhered to a set of “zero COVID” policies for nearly 3 years, such as closing national borders, city-wide lockdown, monitoring “Health Code,” nucleic acid testing, temperature screening, and tracking and quarantining of infected individuals and their direct and indirect contacts (2).

To minimize the impact of the epidemic on economic and social development, on November 11, 2022, the National Health Commission of China issued a “20 Measures policy.” With this policy, some of the strict prevention and control measures were relaxed, only lock-downing the infected areas and buildings, identification of close contacts and infected persons, shortening the period of isolation for medical observation, eliminating the inbound flight fusion mechanism, and large-scale testing and reporting no longer mandatory. Beginning on December 7, 2022, the “20 Measures policy” was replaced with the “10 Measures policy” (3). Under this policy, nucleic acid testing and health codes for inter-regional personnel have been eliminated. Close contacts and infected persons were isolated and quarantined at home. The school conducts offline teaching and ensures normal life. On December 27, 2022, policies of comprehensive liberalization were implemented. A new case of coronavirus infection released from quarantine. Nucleic acid testing and reporting were voluntary. Close contacts and high-risk areas were no longer identified (4). By the end of December 2022, some new sub-variants of Omicron caused the Chinese population a second wave of breakthrough infections in a short time (5). The Chinese Center for Disease Control and Prevention (CDC) reported a positive rate of 29.2% (the number of COVID-19 Nucleic Acid positive tests peaked at 6.94 million), and 2.867 million visits to the doctor for fever (6).

The alteration in learning styles implies that this year’s nursing students face a higher risk during their placements than before the COVID-19 pandemic (7, 8). The nursing curriculum in China comprises 3 years of face-to-face pre-clinical teaching followed by a 1-year internship before the pandemic. Interactive basic nursing skills are normally taught and practiced in every training class. In response to the COVID-19 pandemic, all measures were adopted to reduce the proportion of infected persons. Our educators offered online courses for nursing students instead of in-person learning and interrupted the clinical practice (9).

Earlier studies in the pandemic have reported that nursing students had high levels of distress in their clinical placements, related to the cancelation of clinical placements and transfer to simulated learning (10). The transformation of learning structure and the ways of teaching has resulted in nursing students being exposed to greater risks after the full liberalization of COVID-19 in China. This factor may increase the prevalence of post-traumatic stress disorder (PTSD) caused by COVID-19 pandemic-related experiences (11). After the full liberalization of COVID-19, the SARS-COV-2 omicron variant rapidly accelerated its spread, leading to breakthrough infections in the Chinese population within a short period. Previous research showed that the COVID-19 ongoing stress amplifies negative impacts on student mental health (12). The surge in cases has placed a huge burden on their daily work which might make them vulnerable to psychological problems (12). Studies of health workers at the frontline have shown that increased workload, fear for their health and that of their families and colleagues, lack of personal protective equipment, stigmatization, and caring for patients who have contracted the virus during the pandemic can lead to psychological distress (13, 14). Several studies explored the psychological and mental health impacts of the first wave of the COVID-19 epidemic on nursing students, showing an increase in fear, anxiety, depression, and sleep problems (15, 16).

Participating in nursing internships, they may have to work in a COVID clinical placement and directly care for clinical patients with suspected and confirmed COVID-19. For nursing students, they may fear infection due to a lack of physical preparedness or fear of contagion of the virus to friends and family. When providing hospice services, they may lack the emotional preparedness to witness a patient’s suffering or death. Therefore, these factors may make them more susceptible to PTSD. Posttraumatic stress disorder (PTSD) is a psychiatric disorder that occurs in some people after experiencing a shocking, disturbing, frightening, or dangerous traumatic event. Symptoms of PTSD manifest as intrusiveness (flashbacks or recurrent intrusive thoughts), avoidance (avoidance of the traumatic event that may trigger distressing memories), cognitive and mood changes (negative and distorted beliefs that lead to alienation from others), and altered arousal and reactivity (irritability, reckless behavior, hypervigilance, susceptibility to shock and sleep problems) (17).

Trauma from COVID-19 may cause PTSD among nursing students (11). Nursing students are more likely than other care providers to experience anxiety and psychological stress due to their inadequate skills, inexperience, eagerness to complete their education, uncertainty about the conclusion of the pandemic, and youth-related emotions (18). Studies have found that after a three-month COVID-19 lockdown, nursing students in Spain showed PTSD with a prevalence of 26.7% (19). During the initiation of the COVID-19 pandemic, the prevalence of PTSD among Chinese nursing students was 44.5% (20). Studies in China in 2022 have found that the prevalence of PTSD among nursing students was 15.42% (13). However, to our knowledge, after the full liberalization of COVID-19, the impact has not been evaluated among the intern nursing students in China who were providing health care, when Omicron variant infections spread rapidly and the surge in cases led to another large-scale epidemic. Therefore, the purpose of this study was to examine the prevalence of fear, resilience, and posttraumatic stress disorder (PTSD) and the factors contributing to psychological distress among nursing students entering clinical wards after the full liberalization of COVID-19.

## 2 Materials and methods

### 2.1 Ethical considerations

All participants provided informed consent. The study was approved by the Ethics Committee of the nursing department of Wannan Medical College (approval number 20220004).

### 2.2 Design and participants

#### 2.2.1 Design

The website link has been provided via social platforms (e.g., WeChat and QQ) from 14 January to 19 January 2023. Participants for the study were selected through a snowball sampling process. The professor sent the link (Online surveys are available on the Wen Juan Xing platform) to the head of each class who was willing to distribute it to the students in the class. All participants gave informed consent and understood the purpose of the study. Finally, 438 students completed the survey, and the response rate was 68.87%.

#### 2.2.2 Participants

Participants were recruited from a medical university who are spread across 28 different hospitals in Anhui for internships.

Criteria for inclusion were (1) undergraduate nursing students, (2) voluntary participation in research, and (3) completion of a 9-month internship.

Four hundred and thirty-eight participants completed the survey.

### 2.3 Assessment and evaluation

#### 2.3.1 Demographic factors

The following demographic characteristics were assessed: age, gender (male or female), living area (rural, town, city), monthly household income *per capita* (¥ <2,000, ¥ 2001–6,000, ¥ 6,001–10,000, ¥ >10,001).

#### 2.3.2 COVID-19 and internship-related questions

The following questions were asked with a yes/no answer: Q1. Have you been infected with COVID-19 in the last 6 months? Q2. Have you been isolated in the last 6 months? Q3. Has a friend or relative of yours died as a result of COVID-19 infection? Q4. Are you worried about a second or third wave of viral spikes from COVID-19? Q5. Did you often have direct contact with or care for COVID-19 patients in the hospital during your internship? Q6. Did you experience any negative incidents in the hospital during your internship (e.g., verbal or physical violence)? Q7. Did you have any professional exposure during your internship? Q8. Have you been assigned to work with an experienced care team during your internship?

#### 2.3.3 The fear of COVID-19

Fear of COVID-19 Scale (FCV-19S) was used to assess the COVID-19 fears’ levels of participants (21). This seven-item tool was rated on a five-point Likert scale. One is “strongly disagree” and five are “strongly agree.” The total score (ranges from 7 to 35) is calculated by combining the scores of all items. Coronavirus-19 fear increases with a higher score. A cut-off score of 21 and above has been proposed to differentiate the severity of fears associated with COVID-19 (22).

#### 2.3.4 Resilience

Connor-Davidson Resilience Scale (CD-RISC 10) was used to measure psychological resilience (23). The CD-RISC has 10 self-assessment items, with answers ranging from “0- not true at all” to “4 true nearly all the time.” The maximum total score is 40, calculated from the sum of each question. A threshold score of 30 and above indicated greater resilience to stress. The Chinese version showed good structure validity (24).

#### 2.3.5 PTSD

The Primary Care PTSD Screen for DSM-5 (PC-PTSD-5) was used to examine PTSD symptoms of internship nursing students in Anhui, China after the full liberalization of China’s COVID-19 prevention and control policy (25). The total scores of this 5-item scale ranged from 0 to 5, with two answers for each item (0 = No, 1 = Yes). Above the cut-off value, 3 has been identified as a reference screening for PTSD.

### 2.4 Statistical analysis

Statistical analyses were conducted by using version 21.0 of IBM SPSS (Chicago, IL, United States). Demographic characteristics, COVID-19-related questions, and internship-related questions were described by frequencies and proportions. Chi-Square was used to assess for comparison of categorical variables. A binary logistic regression analysis using the forward stepwise method with the occurrence of PTSD as a dependent variable was performed. Odds ratios (ORs) and 95% confidence intervals (CIs) were used to estimate associations. *p* values were two-tailed and considered to indicate statistical significance at the <0.05 level. The statistical analysis was conducted by Hairong Liu.

## 3 Results

### 3.1 Demographic characteristics

The mean age of the nurse students was 22.11 ± 0.92 years. Of the 438 nurse students investigated in this study, 76.5% were female; 50.9% were from rural, 25.8% were from town, and 23.3% were from city. Most participants (53.2%) had a *per capita* household income range of RMB ¥ 2,001 ~ 6,000 per month (Table 1).

**Table 1:** Sample characteristics and analysis of variables for possible association with PTSD (*N* = 438).

Variables	Characteristics	Total	PTSD *N*(%)		χ^2^	*p*
		*N* (%)	No	Yes		
Gender	Male	103 (23.5)	85 (21.9)	18 (36.7)	5.360	0.021
	Female	335 (76.5)	304 (78.1)	31 (63.3)		
Living area	Rural	223 (50.9)	195 (50.1)	28 (57.1)	2.589	0.274
	Town	113 (25.8)	105 (27.0)	8 (16.3)		
	City	102 (23.3)	89 (22.9)	13 (26.5)		
Household income *per capita*	<2,000	78 (17.8)	70 (18.0)	8 (16.3)	2.516	0.472
	2,001–6,000	233 (53.2)	209 (53.7)	24 (49.0)		
	6,001–10,000	89 (20.3)	75 (19.3)	14 (28.6)		
	>10,001	38 (8.7)	35 (9.0)	3 (6.1)		
Q1. Have you been infected with COVID-19 in the last 6 months?	No	51 (11.6)	44 (11.3)	7 (14.3)	0.374	0.541
	Yes	387 (88.4)	345 (88.7)	42 (85.7)		
Q2. Have you been isolated in the last 6 months	No	337 (76.9)	298 (76.6)	39 (79.6)	0.219	0.640
	Yes	101 (23.1)	91 (23.4)	10 (20.4)		
Q3. Has a friend or relative of yours died as a result of the COVID-19 infection	No	391 (89.3)	351 (90.2)	40 (81.6)	3.359	0.067
	Yes	47 (10.7)	38 (9.8)	9 (18.4)		
Q4. Are you worried about a second or third wave of the pandemic	No	103 (23.5)	98 (25.2)	5 (10.2)	5.436	0.020
	Yes	335 (76.5)	291 (74.8)	44 (89.8)		
Q5. Did you often have direct care for COVID19 patients in hospital during your internship	No	333 (76.0)	307 (78.9)	26 (53.1)	15.967	0.000
	Yes	105 (24.0)	82 (21.1)	23 (46.9)		
Q6. Did you experience any negative incidents in the hospital during your internship (e.g., verbal or physical violence)	No	234 (53.4)	218 (56.0)	16 (32.7)	9.567	0.002
	Yes	204 (46.6)	171 (44.0)	33 (67.3)		
Q7. Did you have any professional exposure during your internship	No	263 (60.0)	247 (63.5)	16 (32.7)	17.256	0.000
	Yes	175 (40.0)	142 (36.5)	33 (67.3)		
Q8. Have you been assigned to work with an experienced team	No	286 (65.3)	261 (67.1)	25 (51.0)	4.962	0.026
	Yes	152 (34.7)	128 (32.9)	24 (49.0)		
The fear of COVID-19	No	364 (83.1)	343 (88.2)	21 (42.9)	63.653	0.000
	Yes	74 (16.9)	46 (11.8)	28 (57.1)		
Resilience	No	370 (84.5)	323 (83.0)	47 (95.9)	5.509	0.019
	Yes	68 (15.5)	66 (17.0)	2 (4.1)		
PTSD	No	389 (88.8)				
	Yes	49 (11.2)				

### 3.2 COVID-19 and internship-related questions

The distribution of COVID-19 and internship-related issues is displayed in Table 1. The majority of the participants (88.4%) tested positive for COVID-19 in the last 6 months. 23.1% of the participants declared that they had been isolated in the last 6 months. 10.7% of the participants had friends or relatives who died as a result of COVID-19 infection. More than three-quarters of the participants (76.5%) worried about a second or third wave of the pandemic. 24% of samples directly cared for patients with COVID-19. Participants reported experiencing verbal or physical violence and occupational exposure during hospital internships in 46.6 and 40% of cases, respectively. One-third reported working with an experienced team.

### 3.3 The level of fear, resilience, and PTSD

The mean score of Fear, Resilience, and PTSD information in Anhui, China was 15.24 ± 4.86, 23.46 ± 5.81, and 0.78 ± 1.24, respectively. The prevalence of fear, resilience, and PTSD was 16.9, 15.5, and 11.2%, respectively. Of the respondents with PTSD symptoms, 63.3% were female, 57.1% were from rural areas, 49% had a monthly *per capita* household income between ¥ 2,001 and ¥ 6,000, 85.7% had tested positive for COVID-19 in the past 6 months; 20.4% of the participants indicated that they had been isolated in the last 6 months, 18.4% of the participants had a friend or relative who had died from COVID-19 infection, 89.8% were concerned about a second or wave 3 pandemic, 46.9% directly cared for COVID-19 patients, 67.3% had experienced verbal or physical violence in hospital, and experienced occupational exposure, 49.0% were assigned to an experienced team, 57.1% were afraid of COVID-19, 95.9% had poor resilience.

### 3.4 Associated factors of PTSD

There were several factors in the research identified to be significantly associated with the occurrence of PTSD through a Chi-square test, including gender (*p* = 0.021), fear of a second or third pandemic wave (*p* = 0.020), direct care of COVID patients in the hospital (*p* = 0.000), the experience of verbal or physical violence at work (*p* = 0.002), the experience of occupational exposure in the hospital (*p* = 0.000), working with an experienced team (*p* = 0.026), fear-19 (*p* = 0.000), and resilience (*p* = 0.019) (Table 1).

The results of the binary logistic regression analysis showed that direct care of COVID patients in hospital (OR = 2.084, 95%CI 1.034 ~ 4.202), the experience of occupational exposure (OR = 2.856, 95%CI 1.436 ~ 5.681), working with an experienced team (OR = 2.120, 95%CI 1.070 ~ 4.198), and fear-19 (OR = 8.269, 95%CI 4.150 ~ 16.479) were significantly and positively associated with a higher risk of PTSD in nursing internship students (Table 2).

**Table 2:** Binary logistic regression analysis of factors influencing PTSD (*N* = 438).

Variables	*B*	SE	Wald	*p*	OR	95%CI
(Constant)	−3.806	0.365	108.646	0.000	0.022	
Direct care of COVID patients in hospital	0.734	0.358	4.214	0.040	2.084	(1.034 ~ 4.202)
Experience of occupational exposure in hospital	1.049	0.351	8.941	0.003	2.856	(1.436 ~ 5.681)
Working with an experienced team	0.751	0.349	4.640	0.031	2.120	(1.070 ~ 4.198)
Fear-19	2.113	0.352	36.057	0.000	8.269	(4.150 ~ 16.479)

## 4 Discussion

Due to the unpredictable nature of epidemics, after COVID-19 was fully liberalized in China, Omicron variant infections spread rapidly and the surge in cases led to another large-scale epidemic. More than 80% of the intern nursing students have been infected, and over 75% of students are worried about another COVID-19 pandemic wave. Therefore, following the full liberalization of China’s COVID-19 prevention and control policy, it is still necessary to know the prevalence of PTSD and its influence factors among intern nursing students.

### 4.1 The prevalence of PTSD

The present study was to assess positive PTSD cases in intern nursing students and to identify associated risk factors after the full liberalization of COVID-19 in China. The prevalence of PTSD was found to be 11.2% among intern nursing students. 16.9% of students expressed fear of COVID-19. The level of PTSD in our sample is lower than those reported by the studies invested in the initial phase of the COVID-19 outbreak, the prevalence in Spain and China was 26.7 and 44.5%, respectively (19, 20). The proportion of the sample reporting lower levels of PTSD than the Australian study (49.4%) (26). Compared to the domestic literature in 2022 (15.42%), the prevalence of PTSD in the study was relatively lower (11.2%) (13). Differential exposure, and the diminishment of symptoms over time, may explain the lower levels of symptom reporting in our sample.

In the international, nursing management is essential to the delivery of care and effective organization (27). During the COVID-19 crisis, nursing managers and leaders are leading the way in responding to the special requirements of their workforce. Nurse managers at all levels are being put to the test in a unique set of circumstances brought about by the response to the COVID-19 epidemic in terms of their practical and ethical judgment (28). To reduce stress for nurses, they need to provide suitable rest days and personal safety measures, arrange support services, rotate allocations of critical patients, and be accessible to staff (29). According to Shahrour’s research, coping with self-efficacy can lessen the impact of psychological stress on nurses’ traumatic experiences and healthcare institutions should provide psychosocial support for nurses (29). Nurse leaders should actively participate in protecting the personal safety of their employees by working closely with hospital administration to ensure and provide personal safety measures (29). At the same time, the leaders or managers themselves need support from other colleagues (30).

### 4.2 Influencing factors on PTSD

Our respondents reported that direct care of patients with COVID-19, the experience of occupational exposure, working with an experienced team, and fear-19 were significantly associated with the severity of PTSD symptoms.

#### 4.2.1 Direct care of COVID patients in hospital

The study found that intern nursing students, as caregivers of COVID-19 patients, hold a positive correlation with PTSD. After the COVID-19 epidemic, medical students may experience higher levels of stress than non-medical students (31). When it comes to primary and secondary infectious disease prevention, nurses are the leading proactive partners (32). A significant risk of infection exists for nursing interns who care for patients in hospitals who have the COVID-19 virus, whether suspected or confirmed. To carry out their jobs, frontline nurses frequently work under pressure and sometimes may even risk their lives (33, 34). The students lacked experience in providing care for those infected with COVID-19, and even experienced nurses required psychological support throughout the pandemic. They have to make difficult choices about patient care in unpredictable and exhausting conditions. For example, inconsistent access to protective equipment, shifting guidelines, high-nurse–patient ratios, and increased workload and hours (35–37). The public was afraid of contracting COVID-19, including friends and family. Some people avoided, harassed, and even blamed medical professionals including nurses and nursing students for spreading COVID-19 (38–40). The possibility of contracting COVID-19 and infecting their families worried the nursing students who worked in the hospital as well (41). Moreover, nurses who provide direct care to COVID-19 patients frequently see patients suffering and death, which may cause them to become emotionally distressed and tired (42). As a result, it may cause a great deal of psychological stress, anxiety, and depression. Formulating long-term strategies and delivery of interventions to address psychosocial problems in intern nursing students are necessary. In Huang’s research, more psychological support, adequate medical protective equipment, training in coping strategies, and developing interventions to block the spread of infectious diseases, could improve the quality of patient care (43).

#### 4.2.2 Experience of occupational exposure in hospital

The study found that experience of occupational exposure in hospitals is another variable, which was positively correlated with PTSD in intern nursing students. Wilson et al. found that first responders experience occupational exposures that are regularly linked to PTSD (44). It was partly in accordance with this research. The occupational exposure to nursing is wide and includes things such as needlestick injuries (45), radiation exposure (46), body fluid exposure (47), and violent workplace environments (48). Of those who had experienced occupational exposure, 67.35% were nursing staff (49). It may related to the fact that nurses need to perform many operations such as oral care, sputum suction, and monitoring vital signs. Liu’ research found similar results that nurses may face a higher risk of occupational exposure to COVID-19 patients (50). It was caused by their increased time spent on wards, direct patient care, and responsibility for gathering sputum for viral testing. Several studies indicated that female are more prone to occupational exposure than their male counterparts (51, 52). The effects of PTSD brought on by occupational exposure can be extremely burdens to individuals, their families, their places of employment, and society at large (53). Compared to individuals without this diagnosis, those with PTSD diagnoses are more likely to report physical illnesses, suicide attempts, poor quality of life, additional mental disorders, and disability (54, 55). Understanding the present state of occupational exposure in intern nursing students will be beneficial in enhancing prevention awareness, preventing occupational exposure, and promoting their physical and mental well-being. To deliver effective education, the teachers need to learn about the characteristics of the nursing student cohort served by the lesson and design the lesson accordingly (56). Distance learning is being added to nursing programs more and more to improve learning opportunities and meet the increased diversity among learners (56). According to Magdalinou’s research, student and teacher interaction is crucial, and an online course with a proper design could facilitate it (57). Moreover, the use of interactive teaching methods would promote learning, and prompt technical support would decrease technological problems and encourage distance learning (57).

#### 4.2.3 Working with an experienced team

It is worth noting that student collaboration with an experienced team is another factor influencing PTSD in intern nursing students. Experienced frontline nurses are more likely to care for infected patients. This result indicates that nursing students working with experienced teams may have higher rates of infection. Studies in southern Iran have shown that nursing students caring for COVID-19 patients face high levels of death anxiety (18). Many studies conducted during the pandemic, focused on the Psychological and mental impact on first-line healthcare workers and showed that the frontline healthcare workers had anxiety, depression, stress, and PTSD (58, 59). After years, they were still fighting with contagion caused by Sars-CoV-2. The study conducted by Claponea RM shows that female gender, not having children, and single marital status, have a high level of burnout Syndrome (60). Burnout can have negative consequences (61). The experienced team was already fatigued and exhausted (62–65). These include reduced quality of care, absenteeism, turnover, and reduced commitment (27). Providing emotional support interventions for intern nursing students is required (66). To reduce anxiety and stress, the faculty should create a structured learning environment, follow the course schedule, communicate changes or updates promptly, modify assignments to fit the learning environment, take self-care, extend grace, and make use of campus, local, and national resources (67).

#### 4.2.4 Fear COVID-19

This study also found that the fear of a second or third wave pandemic was significantly associated with the severity of PTSD symptoms. Because of its deadly features, COVID-19 presented a new worldwide threat (e.g., rapid transmission, uncertainty about the transmission method, incubation period, unpredictable mortality rate, and manifestation), which rapidly evoked anxiety and fear in the Chinese population and worldwide (68). Many countries promote the transition of universities from on-site teaching and learning activities to online or virtual modalities (69). Logically, this COVID-19 epidemic and these preventive measures may have a substantial negative effect on mental health, causing widespread stress, anxiety, depression, and fear (70). The impact of PTSD on intern nursing students is profound, Students have difficulty defining their professional identities, fitting in with the team, and fearing COVID-19. Their psychological conditions were quickly made worse by long-term isolation and uncertainty (71). Even though the majority of students (67.07%) did not actively participate in the treatment of COVID-19 patients, there was still a degree of concern about contracting the virus (72). Compared with adults, students are a more vulnerable population. A recent meta-analysis, which included 33 different researchers from 18 different countries and conducted among university students, confirmed that the fear of COVID-19 was related to PTSD (73). Nursing students are worried about their future professions and study due to the uncertainty about their future careers. They also suffer constant pressure due to factors like separation from family and fear of infection, which raises the possibility of triggering PTSD (74). Consequently, nursing students and educational administrators must recognize the importance of the screening, prevention, and psychological intervention of PTSD. Schools and hospitals should concentrate on teaching nursing students all available coping methods to increase their capacity to control their emotions and effective coping tools to enhance the lives of nursing students, and their families (75).

### 4.3 Strengths and limitations of the study

The research has several strengths. First, this research included 438 participants from different hospitals, which ensured the accuracy of this research. Second, the measures of the fear of COVID-19, resilience, and PTSD were widely applied and validated instruments to thoroughly understand the research questions. Finally, its potential contribution to the field of public health, as it presents the prevalence of PTSD and its influencing factors among intern nursing students after the full liberalization of the COVID-19 prevention and control policy in China.

Several limitations may have an impact on the interpretation of the study. Such as self-report surveys, misinterpretation, and self-report bias could happen. Second, the data was accessed from the participants with social media (smartphones, WeChat, blogs, and computers). Thus, the recruited sample with no Internet was not examined. Third, the interns in this study were from a university in Anhui Province, so the sample cannot be generalized to nursing interns throughout China. In the future, the study will be expanding sample size and conducting multi-center, large-sample studies.

## 5 Conclusion

After COVID-19 full liberalization in China, intern nursing students still experienced pandemic-related mental distress, which can bring PTSD. Adequate support and counseling should be provided, as needed, to intern nursing students who are about to enter the workforce and have experienced severe PTSD symptoms related to COVID-19. Our findings indicated that should understand the importance of screening, formulate intervention strategies and preventive measures to address psychosocial problems and provide coping skills training to intern nursing students.

## Data availability statement

The raw data supporting the conclusions of this article will be made available by the authors, without undue reservation.

## Ethics statement

Written informed consent was obtained from the individual(s) for the publication of any potentially identifiable images or data included in this article.

## Author contributions

YS: Writing – original draft, Writing – review & editing. XianL: Funding acquisition, Writing – original draft, Writing – review & editing. HL: Data curation, Funding acquisition, Methodology, Software, Writing – review & editing. XiaoL: Project administration, Writing – review & editing. LS: Project administration, Writing – review & editing. LZ: Project administration, Supervision, Writing – review & editing. CW: Writing – review & editing. JL: Project administration, Writing – review & editing. ML: Project administration, Writing – review & editing. DZ: Project administration, Writing – review & editing. YL: Project administration, Writing – review & editing. TY: Funding acquisition, Project administration, Supervision, Writing – original draft.

## References

[1] Cucinotta D Vanelli M. WHO declares COVID-19 a pandemic. Acta Biomed. (2020) 91:157–60. doi: 10.23750/abm.v91i1.9397, PMID: 32191675 PMC7569573

[2] Liu W Guan WJ Zhong NS. Strategies and advances in combating COVID-19 in China. Engineering (Beijing). (2020) 6:1076–84. doi: 10.1016/j.eng.2020.10.003, PMID: 33078078 PMC7558233

[3] Xinhua. [Internet]. China Focus: COVID-19 response further optimized with 10 new measures (2022). Available at: https://english.news.cn/20221207/ca014c043bf24728b8dcbc0198565fdf/c.html.

[4] China TNHCotPsRo. [Internet]. Circular on the issuance of a general programme for the implementation of "Type B B control" for novel coronavirus infections. (2022). Available at: https://www.chinacdc.cn/jkzt/crb/zl/szkb_11803/jszl_11815/202212/t20221227_263122.html.

[5] Ge J. The COVID-19 pandemic in China: from dynamic zero-COVID to current policy. Herz. (2023) 48:226–8. doi: 10.1007/s00059-023-05183-5, PMID: 37294456 PMC10252163

[6] Prevention CCfDCa. COVID-19 clinical and surveillance data—December 9, 2022 to April 27, 2023, China [Internet]. (2023). Available at: https://weekly.chinacdc.cn/fileCCDCW/cms/news/info/upload/e712e241-fbcb-426b-9ac8-551ce6fd7ccc.pdf.

[7] Rayan A. Psychological impacts of transition to distance learning due to COVID-19 on nursing students. Int J Ment Health Nurs. (2023) 32:767–77. doi: 10.1111/inm.13139, PMID: 36912457

[8] Fernandez R Green H Middleton R Halcomb E Moxham L. Development and evaluation of the altered student study environment tool: a tool to measure nursing student concerns relating to academic progression during the COVID-19 pandemic. Nurs Educ Perspect. (2022) 43:147–51. doi: 10.1097/01.NEP.0000000000000936, PMID: 35385428

[9] Cao W Fang Z Hou G Han M Xu X Dong J. The psychological impact of the COVID-19 epidemic on college students in China. Psychiatry Res. (2020) 287:112934. doi: 10.1016/j.psychres.2020.112934, PMID: 32229390 PMC7102633

[10] Lovrić R Farčić N Mikšić Š Včev A. Studying during the COVID-19 pandemic: a qualitative inductive content analysis of nursing students’ perceptions and experiences. Educ Sci. (2020) 10:188. doi: 10.3390/educsci10070188, PMID: 36495616

[11] Joseph RA Turner T Lee C Akers SW Whorley E Goodrich C. Impact of COVID-19 on nursing students. J Christ Nurs. (2022) 39:250–7. doi: 10.1097/CNJ.0000000000000951, PMID: 36048598 PMC9444291

[12] Dewart G Corcoran L Thirsk L Petrovic K. Nursing education in a pandemic: academic challenges in response to COVID-19. Nurse Educ Today. (2020) 92:104471. doi: 10.1016/j.nedt.2020.104471, PMID: 32502723 PMC7263267

[13] Zhang D Qin L Huang A Wang C Yuan T Li X. Mediating effect of resilience and fear of COVID-19 on the relationship between social support and post-traumatic stress disorder among campus-quarantined nursing students: a cross-sectional study. BMC Nurs. (2023) 22:164. doi: 10.1186/s12912-023-01319-4 37193966 PMC10185929

[14] Gheshlagh RG Hassanpour-Dehkordi A Moradi Y Zahednezhad H Mazaheri E Kurdi A. Prevalence of psychological disorders among health workers during the COVID-19 pandemic: a systematic review and Meta-analysis. Int J Prev Med. (2023) 14:25. doi: 10.4103/ijpvm.ijpvm_212_21, PMID: 37033279 PMC10080571

[15] Kochuvilayil T Fernandez RS Moxham LJ Lord H Alomari A Hunt L. COVID-19: knowledge, anxiety, academic concerns and preventative behaviours among Australian and Indian undergraduate nursing students: a cross-sectional study. J Clin Nurs. (2021) 30:882–91. doi: 10.1111/jocn.15634, PMID: 33434378 PMC8013450

[16] Mohamed SH. Stress and anxiety among junior nursing students during the initial clinical training: a descriptive study at College of Health Sciences, University of Bahrain. Am J Nurs Res. (2019) 7:995–9. doi: 10.12691/ajnr-7-6-13

[17] Association AP. What is posttraumatic stress disorder (PTSD)? [Internet]. (2021). Available at: https://www.psychiatry.org/patients-families/ptsd/what-is-ptsd.

[18] Mohammadi F Masoumi Z Oshvandi K Khazaei S Bijani M. Death anxiety, moral courage, and resilience in nursing students who care for COVID-19 patients: a cross-sectional study. BMC Nurs. (2022) 21:150. doi: 10.1186/s12912-022-00931-0 35698221 PMC9189788

[19] Mendez-Pinto I Antuña-Casal M Mosteiro-Diaz MP. Psychological disorders among Spanish nursing students three months after COVID-19 lockdown: a cross-sectional study. Int J Ment Health Nurs. (2022) 32:479–89. doi: 10.1111/inm.13086 36330581 PMC9877867

[20] Gao J Wang F Guo S Hu F. Mental health of nursing students amid coronavirus disease 2019 pandemic. Front Psychol. (2021) 12:699558. doi: 10.3389/fpsyg.2021.699558, PMID: 34475837 PMC8407077

[21] Ahorsu DK Lin CY Imani V Saffari M Griffiths MD Pakpour AH. The fear of COVID-19 scale: development and initial validation. Int J Ment Heal Addict. (2020) 20:1537–45. doi: 10.1007/s11469-020-00270-8 PMC7100496 32226353

[22] Liu H Zhou N Zhou Z Tao X Kong Y Zhang M. Symptoms of post traumatic stress disorder and their relationship with the fear of COVID−19 and COVID−19 burden among health care workers after the full liberalization of COVID−19 prevention and control policy in China: a cross-sectional study. BMC Psychiatry. (2023) 23:902. doi: 10.1186/s12888-023-05399-z 38053075 PMC10696867

[23] Campbell-Sills L Stein MB. Psychometric analysis and refinement of the connor–Davidson resilience scale (CD-RISC): validation of a 10-item measure of resilience. J Trauma Stress. (2007) 20:1019–28. doi: 10.1002/jts.20271, PMID: 18157881

[24] Yu X Zhang J. A comparison between the Chinese version of Ego-resiliency scale and Connor-Davidson resilience scale. Psychol Sci. (2007) 30:1169–71. doi: 10.16719/j.cnki.1671-6981.2007.05.035

[25] Prins A Bovin MJ Smolenski DJ Marx BP Kimerling R Jenkins-Guarnieri MA. The primary care PTSD screen for DSM-5 (PC-PTSD-5): development and evaluation within a veteran primary care sample. J Gen Intern Med. (2016) 31:1206–11. doi: 10.1007/s11606-016-3703-5, PMID: 27170304 PMC5023594

[26] Usher Am K Jackson D Massey D Wynaden D Grant J West C. The mental health impact of COVID-19 on pre-registration nursing students in Australia: findings from a national cross-sectional study. J Adv Nurs. (2022) 79:581–92. doi: 10.1111/jan.15478 36453452 PMC9877832

[27] Bodenheimer T Sinsky C. From triple to quadruple aim: Care of the Patient Requires Care of the provider. Ann Fam Med. (2014) 12:573–6. doi: 10.1370/afm.1713, PMID: 25384822 PMC4226781

[28] Newham R Hewison A. Covid-19, ethical nursing management and codes of conduct: an analysis. Nurs Ethics. (2021) 28:82–90. doi: 10.1177/0969733020988316, PMID: 33472524

[29] Shahrour G Dardas LA. Acute stress disorder, coping self-efficacy and subsequent psychological distress among nurses amid COVID-19. J Nurs Manag. (2020) 28:1686–95. doi: 10.1111/jonm.13124, PMID: 32767827 PMC7436502

[30] Antonio MR Rosa María GD. El Liderazgo Pedag Ógico: Competencias Necesarias Para Desarrollar Un Programa De Mejora En Un Centro De Educaci Ón Secundaria. Perspectiva Educacional. (2014) 53:91–113. doi: 10.4151/07189729-Vol.53-Iss.1-Art.127

[31] Ye W Ye X Liu Y Liu Q Vafaei S Gao Y. Effect of the novel coronavirus pneumonia pandemic on medical students’ psychological stress and its influencing factors. Front Psychol. (2020) 11:548506. doi: 10.3389/fpsyg.2020.548506, PMID: 33178063 PMC7591817

[32] Tung YJ Lo KKH Ho RCM Tam WSW. Prevalence of depression among nursing students: a systematic review and meta-analysis. Nurse Educ Today. (2018) 63:119–29. doi: 10.1016/j.nedt.2018.01.009, PMID: 29432998

[33] Mo Y Deng L Zhang L Lang Q Liao C Wang N. Work stress among Chinese nurses to support Wuhan in fighting against COVID-19 epidemic. J Nurs Manag. (2020) 28:1002–9. doi: 10.1111/jonm.13014, PMID: 32255222 PMC7262235

[34] Catton H. Global challenges in health and health care for nurses and midwives everywhere. Int Nurs Rev. (2020) 67:4–6. doi: 10.1111/inr.12578, PMID: 32083728 PMC7165846

[35] Bruyneel A Gallani MC Tack J d'Hondt A Canipel S Franck S. Impact of COVID-19 on nursing time in intensive care units in Belgium. Intens Criti Care Nurs. (2021) 62:102967. doi: 10.1016/j.iccn.2020.102967, PMID: 33162312 PMC7598359

[36] Cohen J YvdM R. Contributing factors to personal protective equipment shortages during the COVID-19 pandemic. Prev Med. (2020) 141:106263. doi: 10.1016/j.ypmed.2020.106263, PMID: 33017601 PMC7531934

[37] LoGiudice JA Bartos S. Experiences of nurses during the COVID-19 pandemic: a mixed-methods study. AACN Adv Crit Care. (2021) 32:14–26. doi: 10.4037/aacnacc2021816, PMID: 33450763

[38] Abdulah DM Mohammedsadiq HA Liamputtong P. Experiences of nurses amidst giving care to COVID-19 patients in clinical settings in Iraqi Kurdistan: a qualitative descriptive study. J Clin Nurs. (2021) 31:294–308. doi: 10.1111/jocn.15909 34152045 PMC8447173

[39] Bhanot D Singh T Verma SK Sharad S. Stigma and discrimination during COVID-19 pandemic. Front Public Health. (2021) 8:577018. doi: 10.3389/fpubh.2020.577018, PMID: 33585379 PMC7874150

[40] Koh D. Occupational risks for COVID-19 infection. Occup Med Oxford. (2020) 70:3–5. doi: 10.1093/occmed/kqaa036, PMID: 32107548 PMC7107962

[41] Lancet T. COVID-19: protecting health-care workers. Lancet. (2020) 395:922. doi: 10.1016/S0140-6736(20)30644-9 PMC7138074 32199474

[42] Alharbi J Jackson D Usher K. The potential for COVID-19 to contribute to compassion fatigue in critical care nurses. J Clin Nurs. (2020) 29:2762–4. doi: 10.1111/jocn.15314, PMID: 32344460 PMC7267232

[43] Huang L Lei W Xu F Liu H Yu L. Emotional responses and coping strategies in nurses and nursing students during Covid-19 outbreak: a comparative study. PLoS One. (2020) 15:e0237303. doi: 10.1371/journal.pone.0237303, PMID: 32764825 PMC7413410

[44] Wilson S Guliani H Boichev G. On the economics of post-traumatic stress disorder among first responders in Canada. J Commun Safety Well-Being. (2016) 1:26–31. doi: 10.35502/jcswb.6, PMID: 37022655

[45] Cho E Lee H Choi M Park SH Yoo IY Aiken LH. Factors associated with needlestick and sharp injuries among hospital nurses: a cross-sectional questionnaire survey. Int J Nurs Stud. (2013) 50:1025–32. doi: 10.1016/j.ijnurstu.2012.07.009, PMID: 22854116 PMC3996454

[46] Andreassi MG Piccaluga E Guagliumi G Del Greco M Gaita F Picano E. Occupational health risks in cardiac catheterization laboratory workers. Circ Cardiovasc Interv. (2016) 9:e003273. doi: 10.1161/CIRCINTERVENTIONS.115.003273, PMID: 27072525

[47] Zhang L Li Q Guan L Fan L Li Y Zhang Z. Prevalence and influence factors of occupational exposure to blood and body fluids in registered Chinese nurses: a national cross-sectional study. BMC Nurs. (2022) 21:298. doi: 10.1186/s12912-022-01090-y 36333812 PMC9636689

[48] Spector PE Zhou ZE Che XX. Nurse exposure to physical and nonphysical violence, bullying, and sexual harassment: a quantitative review. Int J Nurs Stud. (2014) 51:72–84. doi: 10.1016/j.ijnurstu.2013.01.010, PMID: 23433725

[49] Liu H Wang Y He HY Liu LB Zhang Q Chen JL. Experience of comprehensive interventions in reducing occupational exposure to COVID-19. J Infect Public Health. (2021) 14:201–5. doi: 10.1016/j.jiph.2020.12.011, PMID: 33486376 PMC7737508

[50] Liu Z Wu J Shi X Ma Y Ma X Teng Z. Mental health status of healthcare Workers in China for COVID-19 epidemic. Ann Glob Health. (2020) 86:128. doi: 10.5334/aogh.3005 33102148 PMC7546117

[51] Biswas A Harbin S Irvin E Johnston H Begum M Tiong M. Sex and gender differences in occupational Hazard exposures: a scoping review of the recent literature. Curr Environ Health Rep. (2021) 8:267–80. doi: 10.1007/s40572-021-00330-8, PMID: 34839446 PMC8627292

[52] Pappa S Ntella V Giannakas T Giannakoulis VG Papoutsi E Katsaounou P. Prevalence of depression, anxiety, and insomnia among healthcare workers during the COVID-19 pandemic: a systematic review and meta-analysis. Brain Behav Immun. (2020) 88:901–7. doi: 10.1016/j.bbi.2020.05.026, PMID: 32437915 PMC7206431

[53] Torchalla I Strehlau V. The evidence base for interventions targeting individuals with work-related PTSD: a systematic review and recommendations. Behav Modif. (2017) 42:273–303. doi: 10.1177/0145445517725048 28817953

[54] Sareen J Cox BJ Stein MB Afifi TO Fleet C Asmundson GJ. Physical and mental comorbidity, disability, and suicidal behavior associated with posttraumatic stress disorder in a large community sample. Psychosom Med. (2007) 69:242–8. doi: 10.1097/PSY.0b013e31803146d8, PMID: 17401056

[55] Brady KT Killeen TK Brewerton T Lucerini S. Comorbidity of psychiatric disorders and posttraumatic stress disorder. J Clin Psychiatry. (2000) 61:22–32. 10795606

[56] Sowan AK Jenkins LS. Designing, delivering and evaluating a distance learning nursing course responsive to students needs. Int J Med Inform. (2013) 82:553–64. doi: 10.1016/j.ijmedinf.2013.02.004, PMID: 23478139

[57] Magdalinou A Liaskos J Isaakidou M Mantas J. The transition to distance learning in the era of Covid-19 pandemic: the perceptions and experiences of nursing students. Stud Health Technol Inform. (2022) 295:495–8. doi: 10.3233/SHTI220773, PMID: 35773919

[58] Hossain MR Patwary MM Sultana R Browning MHEM. Psychological distress among healthcare professionals during the early stages of the COVID-19 outbreak in low resource settings: a cross-sectional study in Bangladesh. Front Public Health. (2021) 9:701920. doi: 10.3389/fpubh.2021.701920, PMID: 34858914 PMC8632035

[59] Leng M Wei L Shi X Cao G Wei Y Xu H. Mental distress and influencing factors in nurses caring for patients with COVID-19. Nurs Crit Care. (2020) 26:94–101. doi: 10.1111/nicc.12528 33448567

[60] Claponea RM Pop LM Iorga M Iurcov R. Symptoms of burnout syndrome among physicians during the outbreak of COVID-19 pandemic—a systematic literature review. Healthcare. (2022) 10:979. doi: 10.3390/healthcare10060979, PMID: 35742031 PMC9223230

[61] Maslach C. Finding solutions to the problem of burnout. Consult Psychol J Pract Res. (2017) 69:143–52. doi: 10.1037/cpb0000090

[62] Sagherian K Steege LM Cobb SJ Cho H. Insomnia, fatigue and psychosocial well-being during COVID-19 pandemic: a cross-sectional survey of hospital nursing staff in the United States. J Clin Nurs. (2023) 32:5382–95. doi: 10.1111/jocn.15566, PMID: 33219569 PMC7753687

[63] Mary Pappiya E Mubarak Al Baalharith I Arulappan J Missiriya Jalal S Venkatesan K Salem Al Grad H. Stress and burnout among frontline nurses during COVID-19 pandemic in a middle eastern country. Sage Open Nurs. (2023) 9:23779608231185918. doi: 10.1177/23779608231185918 37457617 PMC10345911

[64] Real-Ramírez J Alberto García-Bello L Robles-García R Martínez M Adame-Rivas K Balderas-Pliego M. Well-being status and post-traumatic stress symptoms in health workers attending mindfulness sessions during the early stage of the COVID-19 epidemic in Mexico. Salud Mental. (2021) 43:303–10. doi: 10.17711/SM.0185-3325.2020.041

[65] Cai Z Cui Q Liu Z Li J Gong X Liu J. Nurses endured high risks of psychological problems under the epidemic of COVID-19 in a longitudinal study in Wuhan China. J Psychiatr Res. (2020) 131:132–7. doi: 10.1016/j.jpsychires.2020.09.007, PMID: 32971356 PMC7489269

[66] Härkänen M Pineda AL Tella S Mahat S Panella M Ratti M. The impact of emotional support on healthcare workers and students coping with COVID-19, and other SARS-CoV pandemics – a mixed-methods systematic review. BMC Health Serv Res. (2023) 23:751. doi: 10.1186/s12913-023-09744-6 37443003 PMC10339499

[67] Fitzgerald A Konrad S. Transition in learning during COVID-19: student nurse anxiety, stress, and resource support. Nurs Forum. (2021) 56:298–304. doi: 10.1111/nuf.12547, PMID: 33484171 PMC8014789

[68] Alshehri FS Alatawi Y Alghamdi BS Alhifany AA Alharbi A. Prevalence of post-traumatic stress disorder during the COVID-19 pandemic in Saudi Arabia. Saudi Pharm J. (2020) 28:1666–73. doi: 10.1016/j.jsps.2020.10.013, PMID: 33424259 PMC7783103

[69] Zhai Y Du X. Addressing collegiate mental health amid COVID-19 pandemic. Psychiatry Res. (2020) 288:113003. doi: 10.1016/j.psychres.2020.113003, PMID: 32315885 PMC7162776

[70] Al Sulais E Mosli M AlAmeel T. The psychological impact of COVID-19 pandemic on physicians in Saudi Arabia: a cross-sectional study. Saudi J Gastroenterol. (2020) 26:249–55. doi: 10.4103/sjg.SJG_174_20, PMID: 32496223 PMC7739997

[71] Rubin GJ Wessely S. The psychological effects of quarantining a city. BMJ (Clinical Research Ed). (2020) 368:m313. doi: 10.1136/bmj.m313, PMID: 31992552

[72] Ulenaers D Grosemans J Schrooten W Bergs J. Clinical placement experience of nursing students during the COVID-19 pandemic: a cross-sectional study. Nurse Educ Today. (2021) 99:104746. doi: 10.1016/j.nedt.2021.104746, PMID: 33545565 PMC7803623

[73] Şimşir Z Koç H Seki T Griffiths MD. The relationship between fear of COVID-19 and mental health problems: a meta-analysis. Death Stud. (2021) 46:515–23. doi: 10.1080/07481187.2021.1889097 33641626

[74] Knipe D Evans H Marchant A Gunnell D John A. Mapping population mental health concerns related to COVID-19 and the consequences of physical distancing: a Google trends analysis. Wellcome Open Res. (2020) 5:82. doi: 10.12688/wellcomeopenres.15870.1, PMID: 32671230 PMC7331103

[75] Hamadi HY Zakari NMA Jibreel E Al Nami FN Smida JAS Ben Haddad HH. Stress and coping strategies among nursing students in clinical practice during COVID-19. Nurs Rep. (2021) 11:629–39. doi: 10.3390/nursrep11030060, PMID: 34968338 PMC8608122

